# Supplementary material for: Are carbon emissions trading and green financial instruments synergistic? -Comprehensive quantitative research based on content analysis
Source: PLoS One. 2024 Mar 7;19(3):e0298601. doi: 10.1371/journal.pone.0298601 (PMC10919601; doi:10.1371/journal.pone.0298601)
Supplement: S1 Data — (ZIP) [file pone.0298601.s001.zip › Data/地级市Z_F工作报告.zip!/╔╜╢1⁄2/╚╒╒╒╩╨/└┤╫╘╣1⁄2╓┌║┼í╛┬φ┐╦╩2╛▌═°í┐_2012.docx]

各位代表：

现在，我代表市人民政府向大会报告政府工作，请予审议，并请市政协委员提出意见。

一、本届政府工作回顾

本届政府履职的四年，是我市改革发展进程中极不平凡的四年，我们经历了百年不遇的国际金融危机冲击，经受了节能减排的重大考验，日照发展历史性地进入国家战略。面对挑战与机遇交织的复杂局面，市政府紧紧依靠省委、省政府和市委的坚强领导，依靠市人大、市政协的监督支持，依靠全市人民的共同努力，认真落实科学发展观，坚持一切为了发展、一切为了人民，深入实施“四大战略”、“双轮驱动”，抢抓机遇、攻坚克难、开拓奋进，全市经济社会发展取得了重大成就。

综合实力跨上新台阶。全市生产总值突破千亿元大关，从2007年的629.6亿元增加到2011年的1214.1亿元，年均增长13.4%；人均生产总值达到4.32万元，是2007年的1.6倍。实现境内财政总收入510.2亿元，其中地方财政收入68.5亿元，是2007年的2.4倍。进出口总值从57.6亿美元增加到207.6亿美元，跃居全省第三位，跻身全国外贸百强市。实现规模以上工业增加值727.6亿元，固定资产投资880.7亿元，社会消费品零售总额365.4亿元，分别是2007年的2.2倍、2.4倍和2倍。主营业务收入过亿元的企业发展到342家，利税过千万元的300家，分别增加211家和223家。港口货物吞吐量实现双亿吨跨越，从1.3亿吨跃升到2.5亿吨，相当于四年再造了一个亿吨大港；集装箱运量达到140万标箱，是2007年的3.3倍。金融机构本外币存贷款余额均突破1000亿元，信用总量突破2000亿元，分别是2007年的2.2倍、2.1倍和2.6倍。县域经济实力增强，东港区、岚山区主要经济指标在全省位次实现前移，莒县、五莲县发展步伐加快。

优势特色产业快速成长。三次产业结构由13.7﹕50.1﹕36.2调整为9.2﹕54.4﹕36.4。临港大工业迅猛崛起，日照钢铁精品基地被列入国家钢铁产业调整振兴规划；汽车及零部件加工已形成整车75万辆、发动机48万台、变速箱50万台的产能；大豆加工能力达到600万吨，居全国第二位。四年新增油品加工能力700万吨、浆纸110万吨、木材300万方。战略性新兴产业加速发展，高新技术产业产值年均增长40.7%，有11家企业被列入全省首批战略性新兴产业重点调度名单，省级以上企业技术和工程技术研究中心达到47家，新创国家级、省级名牌产品和驰名商标97件。现代服务业发展势头强劲，物流企业发展到600家，实现增加值115.3亿元；游客接待量达到2451.4万人次，实现旅游总收入151.4亿元，分别是2007年的2倍和2.3倍；加快推进鲁南金融服务中心建设，招商银行、兴业银行、交通银行、中信证券、大华期货和7家保险公司在我市设立分支机构，日照银行在青岛、济南设立分行，新设村镇银行2家。现代农业发展步伐加快，粮食总产连续七年超百万吨，特色农业面积突破100万亩，规模以上农业龙头企业发展到392家，农民专业合作社达到1809家，无公害农产品、绿色食品、有机农产品和农产品地理标志达到291个，创建为出口农产品质量安全示范市。

基础设施建设实现重大突破。“双城一区、轴向拓展、海陆一体、东西联动”的城市发展框架全面拉开，人居环境持续改善，建成区面积达到93.4平方公里、绿化覆盖率达到43.1%，城镇化率达到47.9%。四年投入建设资金231.8亿元，建成了阳光海岸梦幻海滩、游泳馆、城市规划展览馆、植物园、香河公园等精品工程和天然气长输管线、市区集中供热、垃圾污水处理等大型市政公用设施，完成了山东路拓宽改建、北海路建设等一批城市道路工程，改造城中村62个，城市载体服务功能和管理水平明显提升。加快构筑综合交通运输体系，港口集疏运条件更加完备。四年完成港口固定资产投资114.5亿元，建成了岚山港区30万吨级原油码头和深水航道一期、南作业区主航道等港口公用基础设施，新增生产性泊位13个、通过能力5681万吨。争取国家将山西中南部铁路大通道出海口定在日照港，并全面开工建设。新菏兖日铁路电气化改造、枣临铁路、东平铁路和疏港高速公路、334、335、342省道改建工程建成通车，日照至仪征、东明原油输送管道投入运营。日照机场争取工作取得重大突破，被列入国家和省“十二五”机场建设发展规划，成为华东地区仅有的两个、全省唯一新建机场。完成了4座大中型水库和360座小型水库除险加固，新发展节水灌溉19.4万亩，疏浚治理河道200公里。日照水库向市区供水三期、“北水南调”二期和马陵水库并网供水工程竣工投运；220千伏后村、招贤、稍坡输变电工程建成启用。

开放创新展现新局面。日照成为山东半岛蓝色经济区重点开发建设的“两极”、“三组团”之一；国家主体功能区规划把我市列为重点开发区域，日照的战略地位空前提高。日照经济开发区升级为国家级经济技术开发区，日照国际海洋城被列为全省重点打造的三个中外合作园区之一，日照保税物流中心（B型）封关运作，岚山国家级木材贸易加工示范区获准设立，鲁南（日照）国际贸易与航运服务中心建成启用，中韩陆海联运汽车货物运输通道开通，日照国家海洋公园获得批复。接轨青岛进入桥隧同城新时代。招商引资工作成效明显，四年累计招引亿元以上项目511个，实际到账外资16.1亿美元，在我市投资的世界500强企业达到18家。企业上市工作取得新进展，恒宝食品在马来西亚成功上市，新贵科技在齐鲁股权托管交易中心挂牌交易，日照港通过非公开发行股票募集资金26.9亿元。新一轮政府机构改革顺利完成，行政审批事项精简率达到64.1%。农村综合改革不断深化，集体林权制度改革基本完成。在全省率先实现基本药物制度在基层医疗卫生机构和一体化管理的村卫生室全覆盖，药品价格平均降幅达49.8%，门急诊、住院次均费用分别下降42.4%和43.2%。财税、文化等领域的改革扎实推进。

社会事业蓬勃发展。各级各类教育协调发展，城乡免费义务教育全面实现，学前教育和高中阶段教育基本普及，职业教育得到加强，教育教学质量全面提高。实现了全部乡镇有综合文化站，全部行政村有文化大院或文化活动室，农家书屋基本实现全覆盖。创作推出了大型旅游歌舞表演《日出先照》，新编大型历史剧《毋忘在莒》晋京演出并荣获全省新创作剧目一等奖。对县乡村医疗卫生设施进行了扩建改造和设备升级，公共卫生和基本医疗服务体系得到完善。成功举办了十一届全运会日照赛区比赛和2010年中国水上运动会等重大体育赛事。人口目标责任制考核连年获省一等奖。“十一五”主要污染物总量减排任务全面完成，城市环境综合整治定量考核保持全省第一。耕地总量保持动态平衡。公共安全体系不断完善，“平安日照”建设成效显著。实现了全国双拥模范城“四连冠”。妇女儿童、老年人、残疾人、红十字等事业持续发展，外事侨务、对台事务、民族宗教、打击走私、司法仲裁、统计、人防、史志、档案、气象等工作取得新的成绩。

人民群众得到更多实惠。过去四年各级财政用于民生的投入达217.4亿元，年均递增38.9%，完成市级为民办实事重点项目48件。累计新增城镇就业18.5万人，转移农村劳动力12.2万人。城镇居民人均可支配收入达到20098元，农民人均纯收入8756元，分别是2007年的1.5倍和1.6倍。在全省率先实现新农保和城镇居民养老保险国家试点全覆盖，新农保参保率走在全省前列；城镇基本医疗保险覆盖率达100%，新农合参合率达99.6%；城镇五项保险实现市级统筹，覆盖城乡的社会保障体系初步形成。经济适用房货币直补、廉租房补贴、实物配租和公共租赁住房有机衔接的住房保障体系不断完善。在全省率先建立了以城乡低保为基础，临时救助、自然灾害救助和医疗、教育等专项救助为辅助的新型社会救助体系。连续四年向城乡居民免费发放《民生手册》。全面落实各项惠农政策，累计发放各类补贴21亿元。在全省率先实现全部行政村通硬化路，农村自来水普及率提高到92%，农业机械化综合水平达到80.6%，新建改造农房12.4万户，新建农村综合超市367个，建成新型农村社区273个、社区服务中心108处，农民生产生活条件明显改善，农村面貌发生新的变化。

总之，过去的四年是全市经济快速攀升、综合实力显著增强，转型升级进程加快、优势产业迅速崛起，基础设施全面提升、城乡面貌显著变化，各项改革深入推进、对外开放不断扩大，人民生活明显改善、社会事业全面进步的时期。我市相继获得联合国人居奖、中国人居环境奖、国家可持续发展先进示范区、全国绿化模范城市、首届中国法治政府奖、全国社会治安综合治理优秀市、省级文明城市等荣誉。特别是日照成为山东半岛蓝色经济区的重要增长极，区域发展战略上升到国家层面。今天的日照已经站在新的历史起点，展现出更为广阔的发展前景！

2011年是实施“十二五”规划的起步年，经过全市上下的共同努力，经济社会发展实现了良好开局。生产总值增长12.1%，地方财政收入增长23.2%，进出口总值增长55.9%，规模以上工业增加值增长16.1%，固定资产投资增长23.7%，社会消费品零售总额增长17.3%，城镇居民人均可支配收入增长14.5%，农民人均纯收入增长16.7%，居民消费价格指数涨幅控制在4.5%，城镇登记失业率控制在2.27%，人口自然增长率控制在3.25‰。主要做了以下工作：一是抓投资保增长。把大项目建设作为经济工作的重中之重，全年共安排大项目183个，完成投资319.1亿元，岚桥石化200万吨、金石沥青100万吨高档沥青，晨曦石化石油深加工，山东石大科技等18个项目建成投产或试生产，山西中南部铁路大通道累计完成境内投资31亿元。岚山港区30万吨级矿石码头获国家发改委核准，日照机场选址报告已由民航华东局组织专家评审，青日连铁路被铁道部列为重点推进项目。加大招商引资力度，全年招引推进总投资10亿元以上的重大项目48个，实际到位资金121.6亿元，增长22.5%；新批利用外资项目26个，实际到账外资3.8亿美元。二是抓机遇强蓝区。大力实施蓝色经济区“双十•一城”工程，设立了1.5亿元的蓝色产业发展资金，加快推进307个蓝色产业重点项目和160条重大事项，全年实现蓝色经济增加值515.9亿元，占GDP的比重达到42.5%，比上年提高1.2个百分点。与新加坡胜科集团签署了日照国际海洋城合作开发备忘录，国际海洋城概念性总体规划和产业发展规划编制完成，海洋城管理和开发机构组建成立。三是抓转调促升级。加快培育高新技术产业、先进制造业和现代服务业，全年高新技术产业、装备制造业投资分别增长48.5%和61.3%，服务业投资占固定资产投资的比重达到54.4%。开工建设高新技术和战略性新兴产业项目42项，完成投资83亿元，现代派沃泰自动变速箱、海帝电器50万台液晶电视、五征集团年产10万辆中卡等32个项目建成投产。开工建设重点技改项目360项，完成投资231亿元。加大落后产能淘汰和节能改造力度，年度节能减排任务全面完成，被评为2011中国十佳低碳生态城市。四是抓“三农”固基础。粮食总产达到118.9万吨，是我市2000年以来第二个高产年。完成种植业结构调整30万亩，是近年来调整力度最大的一年。完成植树造林24万亩，是近年来造林面积最多的一年。水利基础设施建设投入达到7亿元，完成了潮白河东港段、巨峰河岚山段治理和24座小型水库除险加固任务，建成各类小微型水源工程2078处，新增蓄供水能力1870万方，争取将龙潭沟水库列为国家“烟水工程”扶持项目并开工建设。五是抓民生保和谐。12件为民办实事重点项目全面完成。提高了城乡低保、“五保”供养、企业退休人员基本养老金、失业保险金、最低工资和城乡居民基本医疗保险财政补助标准，发放价格临时补贴770万元。组织132个市直部门、单位和企业，与158户城乡特困家庭开展结对帮扶。新建保障性住房14236套，确定经济适用房补贴对象662户，发放廉租房补贴2317户。完成城中村改造拆迁面积90万平方米。不断完善应急管理体制，重视抓好安全生产和食品、药品安全监管，严厉打击各类违法犯罪活动，社会保持和谐稳定。援疆工作扎实推进，刀郎中学、现代农业灌溉、安居富民工程三个示范项目交付使用。六是抓作风促落实。以开展“三力”建设年活动为契机，努力推动政府工作提速提效。新的市级行政审批服务大厅建成启用，部门入驻率、审批事项进厅率均达到100%，即时办结率、群众满意率分别提高10和14个百分点。制定出台了《关于建立健全依法科学民主决策机制的实施意见》，决策行为更加规范。主动接受人大、政协监督，全年共办理人大代表建议138件、政协委员提案272件，办复率、满意和基本满意率均达到100%。认真落实党风廉政建设责任制，扎实推进惩防体系建设，从严查处违纪违法案件，保持了清正廉洁的良好形象。

各位代表！

过去四年经济社会发展取得的成就，得益于省委、省政府和市委的坚强领导，得益于市人大、市政协和社会各界的监督支持，得益于历届市委、市政府打下的良好基础，是全市人民团结奋斗的结果。在此，我代表市人民政府，向辛勤工作在各条战线上的全市人民，向各级人大代表、政协委员，向各民主党派、工商联、无党派人士、各人民团体，向离退休老领导、老同志，向驻日照人民解放军和武警官兵，向所有关心支持日照建设与发展的社会各界人士和海内外朋友，表示衷心的感谢并致以崇高的敬意！

过去四年我市经济社会发展取得了显著成绩，但前进道路上仍然面临诸多矛盾和问题。主要是：经济总量仍然偏小，综合实力不强，县域经济发展滞后，支撑跨越发展的基础还不牢固；产业结构不尽合理，科技创新能力不强，资源和环境约束日益突出，转方式调结构任务艰巨；城乡发展还不平衡，群众收入还不够高，民生工作存在薄弱环节；有些政府部门服务意识还不强，办事效率不高，软环境有待进一步改善。对这些问题，我们一定高度重视，采取有力措施，认真加以解决。

二、今后五年的奋斗目标

未来五年，是我市抢抓历史机遇，推动科学发展，提升综合实力，加快建设海洋特色新兴城市的关键时期。根据市第十二次党代会和市“十二五”规划纲要精神，今后五年政府工作的总体要求是：高举中国特色社会主义伟大旗帜，坚持以邓小平理论、“三个代表”重要思想为指导，深入贯彻落实科学发展观，以科学发展为主题，以加快转变经济发展方式为主线，以造福日照人民为根本目的，抓住用好国家战略机遇，坚持“双轮驱动”、“三个集约”，深入实施“四大战略”，构建完善“四大体系”，着力打造蓝色日照、幸福日照、平安日照、生态日照、文明日照，全面提升综合实力、创新能力和发展活力，加快建设海洋特色新兴城市，奋力开创率先创新优质发展的新局面。

经济社会发展的主要目标是：

—— 综合实力显著增强。经济增长速度超过全国、全省平均水平，地方财政收入实现翻番，规模以上工业增加值、固定资产投资年均分别增长15%和20%以上。力争经过五年的努力，东港区、岚山区主要经济指标达到全国百强县水平，莒县、五莲县主要经济指标达到全省平均水平。钢铁、石化、汽车产业主营业务收入分别突破1000亿元，海洋装备、浆纸印刷、粮油加工产业分别突破500亿元。港口货物吞吐量超过3.5亿吨，保税港区获准设立。

—— 转方式调结构迈出实质性步伐。现代产业形成较强竞争力，传统产业实现转型升级，高新技术产业、战略性新兴产业和服务业加快发展，三次产业结构调整为6：55：39，蓝色经济比重达到47%。万元GDP能耗降低17%，化学需氧量、氨氮、二氧化硫、氮氧化物排放量分别削减11.8%、11.9%、15.5%和17.3%，林木覆盖率达到40%。

—— 城市化水平明显提高。主城区改造提升和生态文明乡村建设任务基本完成，城镇服务功能进一步增强，主城区建成区面积达到125平方公里、总人口达到115万人，城镇化率达到55%以上。城市污水、生活垃圾无害化处理率分别达到95%和100%。

—— 社会更加文明进步。民主法治建设进一步加强，公共安全体系健全完善，社会事业全面发展，社会管理创新取得实效，公民素质和城市文明程度普遍提高，创建为全国文明城市。

—— 人民生活更加幸福。民生保障体系进一步健全，社会保障水平进一步提高，城乡居民收入与生产总值同步增长，人民更加富裕，物质文化生活明显改善。

各位代表！

展望未来五年，尽管面临着错综复杂的经济形势和日趋激烈的区域竞争，但只要我们深入贯彻落实科学发展观，牢牢把握蓝色经济区规划建设、日照钢铁精品基地建设、参照执行中部地区扶持政策三大机遇，科学谋划，凝心聚力，真抓实干，就一定能把跨越发展的宏伟蓝图变为现实，一个经济发达、文化繁荣、社会和谐、生态文明、人民富裕的新日照，必将呈现在世人面前！

三、2012年的政府工作

今年是新一届政府工作的开局年，我们将迎来党的十八大胜利召开，做好今年工作意义重大，影响深远。我们要深入贯彻省、市党代会精神，牢牢把握主题主线，坚持“稳中求进、好中求快”，着力做好稳增长、强蓝区、调结构、控物价、惠民生、保稳定各项工作，以优异成绩迎接党的十八大胜利召开。经济社会发展的主要预期目标是：生产总值增长12%，地方财政收入增长15%，进出口总值和实际到账外资均增长10%，规模以上工业增加值增长15%，固定资产投资增长20%，社会消费品零售总额增长16%，城镇居民人均可支配收入和农民人均纯收入均增长12%，居民消费价格指数涨幅控制在4%左右，城镇登记失业率控制在3.5%以内，人口自然增长率控制在4.9‰以内。

（一）保持经济平稳较快增长。一是继续把大项目建设作为经济工作的重中之重，强化投资拉动。今年全市共安排大项目200个，年度计划投资310亿元。对大项目建设继续实行领导包保责任制，签约项目抓落地，落地项目抓开工，开工项目抓投产，确保完成年度投资计划，形成一批新的经济增长点。以更大的气魄抓好招商引资，突出产业招商、园区招商、以商招商和专业招商，主动加强与国内外大企业、大集团、大院所的战略合作，争取尽快突破一批大项目，为今后发展积蓄后劲。着力破解土地、资金等瓶颈制约，改进政府部门工作作风，为招商引资和大项目建设营造良好环境。二是积极扩大消费需求。建立健全最低工资标准、职工工资、退休人员基本养老金、失业保险金和城乡低保标准的正常增长机制，提高城乡居民消费能力。深入实施“万村千乡市场”和城乡居民消费直通车工程，大力推进农超、区超对接，年内新建综合性物流配送中心一处，建设改造一批农产品批发市场、农贸市场、社区菜市场、生鲜超市，减少流通环节，降低流通成本，平抑市场物价。大力开展“满意消费惠万家”活动，健全肉类、蔬菜等重点商品流通追溯体系，让群众放心消费。积极拓展信息、文化、家政、医疗保健、体育健身等新兴消费领域。坚定不移地抓好房地产市场调控，增加普通商品房供给，促进房地产业稳定健康发展。三是加大对企业的扶持力度。对60家骨干企业进行重点扶持，突出抓好煤电油运、资金等要素协调保障，确保企业正常运营。全面落实促进非公有制经济和中小微企业发展的有关政策，加强服务体系建设，促进民营企业健康发展。四是强化财政金融支撑保障作用。坚持不懈地抓好财源建设，加强税收征管，调整优化财政支出结构。积极引进市外金融机构，扩大信贷总量，优化信贷结构，加大对实体经济的扶持力度，着力解决中小企业融资难、融资成本高的问题。推动有条件的企业上市或到场外市场挂牌交易，年内重点扶持10家拟上市企业。发挥山东蓝色经济区产权交易中心的作用，为非上市公司股权托管与交易、公共资源市场化配置等提供平台。

（二）毫不放松地做好“三农”工作。一是加快发展现代农业。大力实施农业十大产业振兴规划和稳定百万吨粮食生产能力建设规划，稳步扩大特色农业种植面积，大力发展现代渔业和畜牧业，年内完成农业结构调整10万亩，粮食总产保持100万吨以上。发展壮大农业龙头企业，做大做强农产品精深加工产业。规范农民专业合作社发展，提高农民进入市场的组织化程度。加强基层农业科技队伍建设，加大农业科技创新力度，推进高产创建、标准化创建和现代农业示范区建设，提高农业机械化水平。二是加强以水利为重点的基础设施建设。重点抓好龙潭沟水库建设，对2座中型水库和29座小型水库进行除险加固，完成莒县袁公河、潮白河五莲段、傅疃河开发区段、巨峰河涛雒段治理任务，争取开工建设“沭水东调”工程。大力建设“旱能浇、涝能排”的高标准农田，完成600处小微型水利设施建设，发展节水灌溉7.4万亩。实施农村集中供水或管网延伸工程，解决13万人的饮水安全问题。加快农村电网改造升级，建设220千伏石井、110千伏南湖等10项输变电工程。三是加快小城镇建设。着力推进城乡规划、产业发展、基础设施和公共服务“四个一体化”，落实扩权强镇措施，重点扶持10个国家重点镇和省级中心镇，支持经济薄弱乡镇加快发展。积极推进户籍制度改革，放宽进城落户条件，引导农民向城镇有序转移。加大村镇基础设施投入力度，实施“农村公路网化示范县”工程，投资4.1亿元改造农村公路1450公里；抓好592个村的垃圾污水处理工程建设。四是突破发展县域经济。把壮大县域经济实力作为一项紧迫任务，树立全域发展理念，推动临港产业和基础设施向区县延伸，引导各区县积极对接三大机遇，突出县域特色，着力抓好大项目带动、产业集群培育、园区建设、城乡统筹四个重点，奋力实现进位争先。

（三）打好转方式调结构的主动仗。一是调强优势主导产业。依托港口优势，大力发展现代港口经济，着力打造集货物吞吐、仓储物流、加工制造、大宗商品交易等于一体的完整产业链条，推动产业向价值链高端延伸，提升综合效益。把日照钢铁精品基地建设作为头号工程，全力推进项目拆迁、填海、相关规划调整、落后产能淘汰、企业重组等各项工作，争取年内开工建设。精心规划配套产业发展，积极推动钢铁与汽车、海洋先进装备制造等产业的结合，拉长产业链条。加快亚太森博三期、威亚发动机三工厂、派沃泰变速箱二工厂、岱摩斯车桥、东方重工海洋装备、凌云海120万吨精炼糖等重点项目建设。二是调快高新技术和战略性新兴产业。积极培育新能源、新信息、新材料、新医药、海洋开发及环保产业，重点抓好活点物联网络、众山生物科技、鲁信金禾、海汇环保机械等52个战略性新兴产业项目。大力发展电子信息、软件和物联网等高新技术产业，加快建设智能工业、智能物流和智能电网，打造数字日照、智慧城市。三是调高服务业。坚持扩大总量与优化结构并举，突出重点城区、重点园区、重点企业、重点项目“四大载体”，优先发展生产性服务业，加快发展生活性服务业，扩大农村服务业规模，推动服务外包、中介服务等新型产业发展，提高服务业比重和水平。大力培育临港物流业，抓好中铁物流加工基地、凌云海物流、临港商务区等重点项目建设，加快打造区域性国际航运物流中心。整合海、山、泉、林、古等资源，提升观光旅游和民俗旅游，培育休闲度假旅游，抓好温泉度假等重点项目开发建设，推动旅游产业上档升级，年内实现旅游总收入177亿元，增长17%。

（四）提升城市基础设施承载能力。一是高水准推进城市规划建设。完成城市总体规划和开发区、高新区、北部旅游度假区控制性详规修编，编制地下空间开发利用和卧龙山风景区规划。高标准、高质量地抓好中心商务区、奥林匹克水上公园北部开发、阳光海岸综合整治、绿道网建设、老城区人居环境改造提升、东关北路商业街建设等10项城建重点工程，新增城市绿化面积91万平方米。建成市供水水质检测中心，提高市区供水质量。抓好淄博路等7条城市道路建设。进一步优化公交线路，完成300处公交候车亭改造升级。加大综合执法力度，提高城市管理水平。二是加强综合交通运输体系建设。抓好岚山港区30万吨级原油码头扩建、30万吨级矿石码头和石臼港西区四期等18项港口重点工程，完成港口货物吞吐量2.82亿吨、集装箱运量170万标箱。全力推进日照机场前期工作，争取早日开工建设。加快推进山西中南部铁路大通道、坪岚铁路扩能改造、胶新铁路电气化改造和204国道拓宽改造工程，争取尽快开工建设潍日、岚临高速公路，积极推进青日连铁路、日照—濮阳—洛阳输油管线前期工作。

（五）大力推进开放创新。一是突出抓好载体建设。全力推动日照经济技术开发区跨越发展，推动山海天旅游度假区向国家级旅游度假区迈进，加快大学科技园转型升级，聚力突破市北经济开发区、高新技术产业开发区，积极争取设立日照保税港区。按照“两心一岛十组团”的布局，加快国际海洋城规划建设。二是保持对外经贸稳步发展。积极应对全球市场需求低迷的挑战，大力实施市场多元化战略，巩固扩大传统市场，拓展新兴市场。深入实施科技兴贸和以质取胜战略，抓好外贸转型升级示范基地和科技兴贸创新基地建设，推动出口产品转型升级，提高机电产品出口比重。支持有条件的企业到境外开展加工贸易和矿产资源开发，承揽境外承包工程。三是深化重点领域和关键环节的改革。抓好鲁南临港产业区综合配套和蓝色经济区发展改革试点工作，发挥先行先试作用。启用市级公共资源交易中心，搭建工程招投标、政府采购、国有土地出让等公共资源交易的阳光平台。深入推进医药卫生体制改革，加快公立医院综合改革，全面落实基本药物配备使用和对乡村医生的补偿政策，促进基本公共卫生服务均等化。积极稳妥地搞好事业单位分类改革。继续深化农村信用社改革。稳定完善农村基本经营制度，稳妥推进农村土地承包经营权流转市场建设。深化水利体制改革，加强基层水利服务体系建设。四是提高科技创新能力。突出企业的科技创新主体地位，鼓励企业加大研发投入，建立自主创新平台，开展产学研联合，加快科技成果转化应用，年内新创20家市级以上企业技术中心。创新人才工作机制，启动蓝色经济引智试验区建设，加快引进紧缺人才和研发团队。大力实施品牌带动战略，加快培育名牌产品和知名品牌。

（六）加快“生态日照”建设。一是建设环境友好型社会。坚持环境生态、产业生态、生活方式生态的理念，以对人民、对历史高度负责的态度，切实保护好日照的蓝天、碧海、金沙滩。坚持尊重自然、敬畏自然，加大对沙滩、岸线、山体、森林、水源等自然生态资源的保护力度，加快修复受损生态环境。切实加强海洋环境保护，高标准规划建设日照国家级海洋公园。下大气力推进节能减排，加强工业、建筑、交通、公共机构等领域用能管理，加大差别电价、惩罚性电价实施力度，抓好落后产能淘汰工作。扎实推进污染物总量减排，抓好现有污染源治理，建设餐厨垃圾处理厂、第三污水处理厂。加强大气污染治理和监测预警，将PM2.5纳入常规空气质量监测体系。二是建设资源节约型社会。实行最严格的耕地保护和集约节约用地制度，用好增减挂钩政策，盘活闲置和低效利用土地，实行行业用地定额和投资强度控制标准，提高单位土地投资强度和产出效益。坚持绿色发展理念，积极推行清洁生产。大力发展循环经济，支持资源综合利用。实行最严格的水资源管理制度，鼓励发展节水型经济，建设节水型社会。

（七）扎实开展“文化建设年”活动。一是加强社会主义精神文明建设。大力宣传社会主义核心价值观，弘扬爱国主义、集体主义、社会主义思想。加强“四德”建设和诚信教育，用社会主义荣辱观引领社会风尚。积极培育城市人文精神，深入开展文明创建活动，争创全国文明城市。二是大力发展公益性文化事业。深入实施千百重点基层文化工程，推进文化惠民项目进镇村进社区，完善100个乡镇、村居综合文化站和文化活动场所，大力开展群众文化活动。加快建设市级综合文化中心，启动建设日照海洋科普馆。深入推进文化体制改革，加强文化市场综合管理，做好第十届中国艺术节有关筹备工作。三是推动文化产业跨越发展。认真落实全市文化产业发展规划，积极培育文化创意、动漫制作、印刷复制、数字内容等新兴文化产业，加快创意产业园、海洋文化产业园、动漫产业园和印刷工业园建设。抓好文化产业示范基地培育，提升黑陶、绿茶、农民画等传统文化产业品质。落实财政投入增长机制，鼓励社会资本有序进入文化产业领域。

（八）切实保障和改善民生。继续加大民生投入，抓好11件为民办实事重点项目，让人民群众得到更多的实惠。一是多渠道扩大就业。积极落实就业扶持政策，努力增加就业岗位，扎实推进基层公共就业服务平台建设，加强职业培训，年内新增城镇就业2.8万人，农村劳动力转移就业1.6万人。二是提高社会保障水平。大力推进城乡居民、个体私营经济和灵活就业人员参加社会保险，把“五七工”、“家属工”和未参保集体企业退休人员纳入基本养老保险范围。将城镇低保标准提高到城镇居民人均可支配收入的20%左右，农村低保标准由1400元提高到2000元，增加保障人数1.2万人。新农合与城镇居民医疗保险补助标准提高到每人每年240元。建设各类保障性住房1.5万套，新增发放廉租住房补贴1105户。加大社会救助力度，扩大城乡特困家庭结对帮扶范围。三是大力发展社会事业。加大教育投入，确保教育支出占财政支出的比重达到20%以上。落实学前教育三年行动计划，新改扩建公办幼儿园39处。加快普通中小学标准化建设，提升教师队伍整体素质，推动义务教育均衡发展。促进普通高中优质特色发展，提高职业教育服务经济建设能力。实施中小学校舍安全工程，加强校车管理和校园安全工作。推进公共卫生体系建设，扶持中医药事业发展，做好重点传染病防控工作，强化药品安全监管，保障人民群众身体健康。积极开展全民健身运动，提高竞技体育水平。严格落实人口目标管理责任制，稳定低生育水平。做好“双拥共建”、优抚安置和人民防空工作，加强国防动员、民兵预备役建设。扎实开展对口援建新疆麦盖提县工作。四是加强和创新社会管理。抓好全国农村社区管理和服务创新实验区试点，提升城乡社区建设水平。扎实推进“六五”普法和依法治市工作，积极推行仲裁法律制度。强化社会治安综合治理，深入开展严打整治斗争。坚决纠正损害群众利益的行为，从源头上预防和减少社会矛盾。建立食品安全综合协调机制，构建全过程、无缝隙的监管体系。严厉打击走私贩私、制假售假等不法行为。深入开展“安全管理提升年”活动，加强应急救援能力建设，坚决遏制重特大事故，确保安全生产形势稳定。

四、加强政府自身建设

立足新起点，实现新跨越，必须进一步加强自身建设，努力打造服务政府、法治政府、效能政府、廉洁政府。

一是坚持执政为民，建设服务政府。政府各部门要切实增强服务意识，为基层、为群众、为企业、为项目提供主动服务、便捷服务、高效服务、全程服务。严格实行行政问责，对吃拿卡要、刁难企业、不作为、乱作为、破坏发展环境的人和事，要发现一起查处一起，决不姑息迁就。充分发挥市长热线电话、市长信箱、行风在线、社情民意调查中心等平台的作用，进一步畅通民意渠道，及时解决群众反映的困难和问题。各级领导干部要深入基层调查研究，问政于民、问需于民、问计于民，以爱民为民的实际行动赢得群众的信任和支持。

二是坚持依法行政，建设法治政府。依法执行人大及其常委会的决议、决定，主动接受人大的法律监督和政协的民主监督，认真做好人大代表建议和政协委员提案办理工作，及时听取各民主党派、工商联、无党派人士、各人民团体和人民群众的意见建议。严格执行《山东省行政程序规定》，扎实开展“行政程序年”活动。完善行政复议机制，推行相对集中行政复议职权。认真落实专家咨询论证、合法性审查、风险评估、社会公示与听证等制度，加强对重大决策的跟踪问效，提高依法科学民主决策水平。积极推进财政预决算和“三公”经费公开，确保权力在阳光下运行。

三是坚持务实高效，建设效能政府。大兴求真务实之风，说实话、办实事、求实效，多做打基础、利长远的事，不搞徒有虚名的花架子。少开会、开短会、讲短话，腾出更多的时间和精力解决实际问题。切实增强抓落实的紧迫感和责任感，说了干，定了办，提高政府执行力。大力推进政府工作提速提效，进一步压减审批事项，推进制度创新、流程再造和网上审批，提高办事效率。牢固树立全局观念，克服推诿扯皮现象，确保政令畅通。健全重大决策部署执行情况检查和专项督查制度，坚决反对以会议贯彻会议、以文件落实文件，确保各项工作任务落到实处。

四是坚持清廉节俭，建设廉洁政府。深入开展党风廉政建设和反腐败斗争，严格执行党风廉政建设责任制，扎实推进惩防体系建设，从严查处违纪违法案件。大力推进专项治理工作，坚决纠正群众反映强烈的不正之风。落实建设工程招投标、经营性土地招拍挂、政府采购和“收支两条线”等制度，严格政府投资项目、公共资金和事业单位经营性收入管理，推进政府部门经营性资产分离改革，从源头上预防腐败。加大监察、审计监督力度，行政权力运行到哪里、监督就跟踪到哪里；财政资金运用到哪里、审计就跟进到哪里。大力倡导艰苦奋斗的优良作风，勤俭办一切事业，大力压减公用经费支出，降低行政成本，建设节约型机关。

各位代表！

面对千载难逢的重大机遇，站在跨越发展的历史节点，我们倍感责任重大、使命光荣。让我们紧密团结在以胡锦涛同志为总书记的党中央周围，高举中国特色社会主义伟大旗帜，深入贯彻落实科学发展观，在市委的坚强领导下，开拓进取，真抓实干，为加快建设海洋特色新兴城市，创造日照人民更加幸福美好的生活而努力奋斗！
